# Genome-wide analysis of the *CalS* gene family in cotton reveals their potential roles in fiber development and responses to stress

**DOI:** 10.7717/peerj.12557

**Published:** 2021-11-30

**Authors:** Jiajia Feng, Yi Chen, Xianghui Xiao, Yunfang Qu, Pengtao Li, Quanwei Lu, Jinling Huang

**Affiliations:** 1 College of Agriculture, Shanxi Agricultural University, Taigu, Shanxi, China; 2 School of Biotechnology and Food Engineering, Anyang Institute of Technology, Anyang, Henan, China

**Keywords:** *Callose synthase*, Synteny, Gene family, *Gossypium*, Various stresses

## Abstract

Callose deposition occurs during plant growth and development, as well as when plants are under biotic and abiotic stress. *Callose synthase* is a key enzyme for the synthesis of callose. In this study, 27, 28, 16, and 15 *callose synthase* family members were identified in *Gossypium hirsutum*, *Gossypium barbadense*, *Gossypium raimondii*, and *Gossypium arboreum* using the sequence of *Arabidopsis callose synthase*. The *CalSs* were divided into five groups by phylogenetic, gene structure, and conservative motif analysis. The conserved motifs and gene structures of *CalSs* in each group were highly similar. Based on the analysis of cis-acting elements, it is inferred that *GhCalSs* were regulated by abiotic stress. WGD/Segmental duplication promoted the amplification of the *CalS* gene in cotton, and purification selection had an important function in the *CalS* family. The transcriptome data and qRT-PCR under cold, heat, salt, and PEG treatments showed that *GhCalSs* were involved in abiotic stress. The expression patterns of *GhCalSs* were different in various tissues. We predicted that *GhCalS4*, which was highly expressed in fibers, had an important effect on fiber elongation. Hence, these results help us understand the role of *GhCalSs* in fiber development and stress response.

## Introduction

Callose is a linear homopolymer composed of β-1, 3-linked glucose, widely found in higher plants as an important part of specialized cell walls or cell wall-associated structures (Chen & Kim, 2009). However, Callose synthesis mainly depends on *callose synthase* (*CalS*) (Hong, Delauney & Verma, 2001). In *Arabidopsis thaliana*, callose activity was significantly increased and deposited after overexpression of the *AtCalS12* (Ellinger et al., 2013). In *rice*, callose deposition in the plasmodesmata of *crr1* (*AtCalS10* homologous gene) mutants was reduced (Song et al., 2016).

Callose can regulate the transport of plasmodesmata and phloem, affect plant development and response to biotic stress by controlling *callose synthases*. For example, the *AtCalS5* could maintain the normal formation of callose walls during pollen development (Shi et al., 2015a; Shi et al., 2015b). Meanwhile, *CalS* was regulated by various signaling pathways, and different biological regulatory processes involve hormones, transcription factors. For instance, external application of ABA can increase the activity of callose synthase of rice, promoting callose deposition, and thus increasing its resistance to BPH insects (Liu et al., 2017). The expression of *CalSs* were also affected by developmental and stress conditions, such as pollen development (Toller et al., 2008; Xie, Wang & Hong, 2010; Huang et al., 2009; Shi et al., 2015a; Shi et al., 2015b), low-temperature stimulation (Fromm et al., 2013), mechanical wounding (Cui & Lee, 2016; Jacobs et al., 2003; Xie et al., 2011), fungal diseases (Dong et al., 2008; Oide et al., 2013; Ellinger et al., 2014; Blümke, Somerville & Voigt, 2013), bacterial diseases (Granato et al., 2019; Enrique et al., 2011), and insect diseases (Ahmad et al., 2011; Koh et al., 2012; Sun et al., 2018; Yao et al., 2020).

Due to the importance of callose, the *callose synthase* gene family has been identified in various plants. To date, it has been reported as 12 *AtCalSs* in *Arabidopsis thaliana* (Hong, Delauney & Verma, 2001), 12 *CsCalSs* in *Citrus sinensis* (Granato et al., 2019), 32 *BnCalSs* in *Brassica napus* (Liu, Zou & Fernando, 2018), eight *VvCalSs* in *Vitis vinifera* (Yu et al., 2016), seven *HvCalSs* in *Hordeum vulgare* (Schober et al., 2009), 15 *BraCalSs* in *Chinese cabbage* (Pu et al., 2019). Generally, according to the evolutionary analysis of *CalSs* in the above species, the *CalS* family can be divided into three of four main groups (Chen & Kim, 2009; Liu, Zou & Fernando, 2018).

Cotton is an important economic crop in China which yield is affected by biotic and abiotic stresses, producing prevalent fibers for textile industry (Wang et al., 2020; Fang et al., 2014). Some researches have reported that cotton fiber elongation was related to callose deposition which may be involved in the closing of plasmodesmata, then promoted the fiber length (Ruan et al., 2004). It is possible that callose affects fiber elongation by controlling *CalS*. In light of the above, the *CalS* may play a significant role in cotton in responsing various stresses and promoting fiber elongation. A *callose synthase* gene, *CFL1* was identified (Cui et al., 2001), however, the *callose synthase* gene family members, phylogenetic relationships and expression patterns in cotton are still unclear.

In the current study, we identified *callose synthase* genes in two cultivated allotetraploids cotton, *G. hirsutum* and *G. barbadense*, and their two putative genome donors, *G. raimondii* and *G. arboreum*, then discussed their phylogenetic relationships, conserved domains, gene structures, synteny, and cis-acting elements. We also focused on the expression patterns of *GhCalSs* in various tissues and their expression under abiotic stress. These findings provide a solid foundation for further study of the roles of *CalSs* in cotton fiber development and stress responses.

## Materials & Methods

### Plant materials and treatments

Upland cotton TM-1, planted in Anyang Institute of Technology, was subjected to salt stress (350 mM NaCl) and drought stress (12% PEG6000) when the seedling reached two weeks. The leaves were collected 0 h, 1 h, 3 h, 6 h, 12 h, and 24 h after treatment. CCRI45 and MBI7747 were planted on farms managed by Cotton Research of Chinese Academy of Agricultural Sciences in Anyang. Cotton fibers were collected at 5, 10, 15, 20, 25 days post-anthesis (DPA). All samples were stored at −80 °C.

### Identification of *CalS* family members in *Gossypium spp*

The genome sequences and annotated files of *G. hirsutum* (Hu et al., 2019), *G. barbadense* (Hu et al., 2019), *G. raimondii* (Paterson et al., 2012), and *G. arboretum* (Du et al., 2018) were downloaded from Cottongen (https://www.cottongen.org/) (Yu et al., 2014). Both blast and HMMER were used to identify the *CalS* sequences. The 1,3 beta-glucan synthase (PF02364) and FKS1_DOM1 domain (PF14288) from the Pfam database (http://pfam.xfam.org/) were searched by the HMMSearch program in TBtools to determine the presumed protein sequence (Chen et al., 2020). Besides, 12 *Arabidopsis CalSs* (Hong, Delauney & Verma, 2001) were used as queries sequences to identify family members using the Blastp program of TBtools (Chen et al., 2020). The protein sequences without above two domains were rejected and the domain which incomplete were also deleted. Finally, the final sequences were calculated by using ExPASy (https://www.expasy.org/) to calculate the theoretical isoelectric points (pI) and molecular weights (MW) and using the CELLO (http://cello.life.nctu.edu.tw/) for subcellular localization prediction (Yu et al., 2006).

### Phylogenetic tree construction, gene structure, and motif analysis

The phylogenetic tree among four *Gossypium* species and *Arabidopsis thaliana* was constructed by MEGA7 (Kumar, Stecher & Tamura, 2016). It was constructed by the neighbor-joining (NJ) method, with 1,000 bootstrap replicates, then was drawn by using EvolView (He et al., 2016). TBtools was used to extract the location information of *CalSs* and visualize the gene structure. MEME (https://meme-suite.org/meme/tools/meme) was used to identify the conservative motif with the parameter set to the maximum number of motifs: 20.

### Chromosome location and synteny analysis for *CalSs*

The locations of *CalSs* on chromosomes were shown by TBtools using four cotton species genomic annotation files (Chen et al., 2020). MCScanX was used to analyze the collinearity of the *CalSs*, that is, using *CalS* protein sequences to analyze the orthologous and paralogous gene pairs (Chen et al., 2020). Collinear gene pairs were visualized by using the circos (Chen et al., 2020). To investigate the selection pressure between homologous genes, we calculated the nonsynonymous substitutions rate (Ka) and synonymous substitutions rate (Ks) of homologous genes by KaKs_Calculator (Wang et al., 2010).

### Analysis of Cis-acting element in promoters and functional enrichment analysis

The 2,000 bp sequence upstream of the translation initiation codon ATG of *CalS* gene was selected as promoter. The cis-acting elements contained in the promoter region of the *CalSs* were predicted using the PlantCare website (Lescot et al., 2002). For functional enrichment analysis, gene ontology (GO) analysis was performed using the OmicShare tool (https://www.omicshare.com/tools).

### *GhCalSs* expression patterns under different tissues and abiotic stresses

In order to analyze the expression of *GhCalSs* in different tissues and under stress, we downloaded 11 tissues (bract, pental, torus, root, leaf, stem, pistil, sepal, anther, ovule, fiber) and abiotic stress treatment (cold, heat, drought, salt) data from Cotton Omics Database (http://cotton.zju.edu.cn) (accession number: PRJNA490626) (Hu et al., 2019). *GhCalSs* with FPKM > 1 were considered as expressed genes. The expression patterns of the *GhCalSs* were visualized by ComplexHeatmap (Gu, Eils & Schlesner, 2016) based on the value of log_2_(FPKM+1).

### RNA isolation and qRT-PCR analysis

FastPure Plant Total RNA Isolation Kit (RC401, Vazyme) was used to extract RNA, and then we used 1µg to synthesize cDNA (HiScript III 1st Strand cDNA Synthesis Kit, R312 Vazyme). ChamQ Universal SYBR qRT-PCR Master Mix (Q711, Vazyme) was used for qRT-PCR in ABI 7500 Fast Real-time PCR System (Applied Biosystems, USA). Gene-specific primers for qRT-PCR were designed by using primer-blast in NCBI, with melting temperatures of 55–60 °C, product lengths of 101–221 bp, primer length of 18–25 bp (Table S1). For qRT-PCR, the reaction contains 10 µL 2x ChamQ Universal SYBR qPCR Master Mix, 0.4 µL of each primer, 3 µL template, and ddH_2_O to make up the total 20 µL volume. Then it was carried out in the following condition: one cycles of 95 °C for 30 s, 40 cycles of 95 °C for 10 s and 60 °C for 30 s. Each experiment was repeated three times, and two of the completed data were selected for drawing. Expression of all genes were calculated using a 2^−ΔΔCt^ method (Livak & Schmittgen, 2001).

## Results

### Identification and characterization of *CalSs* in *Gossypium spp*

Through the analysis of the *CalS* protein sequences in *Arabidopsis thaliana*, we found that all the protein sequences contain 1, 3-β-glucan synthase (PF02364) and FKS1_DOM1 domain (PF14288), total of 27 members of the *CalS* gene family in *G. hirsutum*, 28 in *G. barbadense*, 15 in *G. raimondii*, and 16 in *G. arboretum* were identified, all of which were named according to their chromosomal locations. The properties of *CalSs* in cotton were further analyzed (Table S2). The protein sequence length of *CalSs* ranged from 1,494 to 1,979 amino acids, with an average MW of 212.88 kDa, and shared high similarity to the *Arabidopsis thaliana CalS* proteins (Hong, Delauney & Verma, 2001). The isoelectric point (PI) values of the above genes were all greater than 7, indicating that *CalSs* in cotton were alkaline, which was the same as the biochemical properties of the *CalSs* in *Chinese cabbage* and *Brassica* (Pu et al., 2019; Liu, Zou & Fernando, 2018). The *CalSs* were most likely localized in the plasma membrane, as predicted in *Arabidopsis thaliana* and *Chinese cabbage* (Pu et al., 2019; Zavaliev et al., 2011).

### Classification and phylogenetic analysis of the cotton *CalSs*

**Figure 1:**
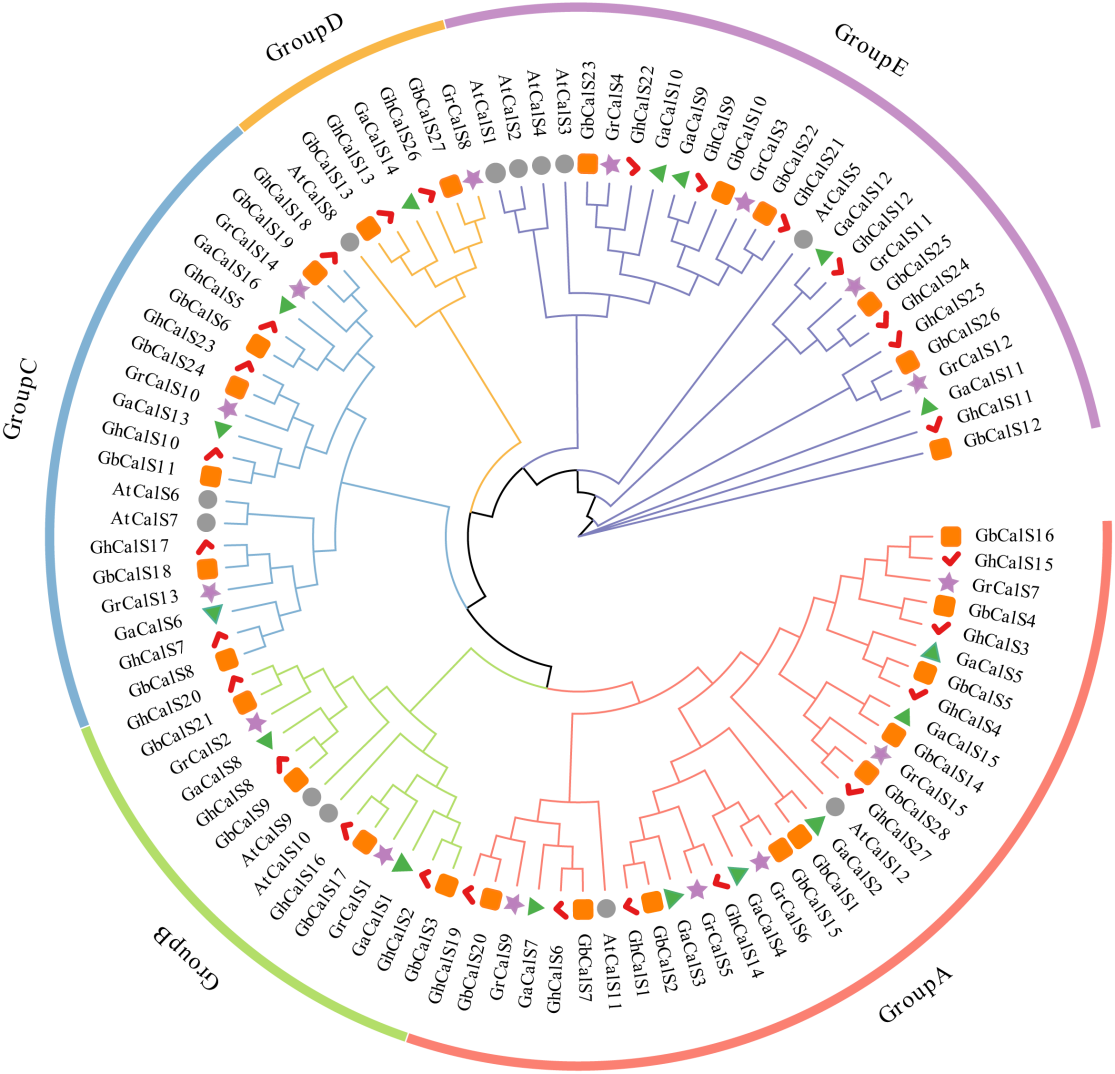
Phylogenetic analysis of *CalS* protein from *G. hirsutum*, *G. barbadense*, *G. raimondii*, *G. arboretum* and *Arabidopsis*. The bootstrap values are shown at the nodes. The *CalSs* from *G. hirsutum*, *G. barbadense*, *G. raimondii*, *G. arboretum*, and *Arabidopsis* are marked with red check, orange rect, purple star, green triangle, grey circle, respectively.

In order to investigate the evolutionary relationships of the *CalSs* in the four cotton species and its relationship with *Arabidopsis thaliana*, a phylogenetic tree was constructed using the protein sequences of *CalSs* (Fig. 1). Based on the phylogenetic tree of this study, the *CalSs* were divided into five groups. The distribution of *CalSs* in each group was shown in Table S3. The members of Group A were homologous to *AtCalS11/AtCalS12*, the members of Group B were homologous to *AtCalS9/AtCalS10*, the members of Group C were homologous to *AtCalS6/AtCalS7*, the members of Group D were homologous to *AtCalS8*, and the members of Group E were homologous to *AtCalS1-5*. There were *Arabidopsis* genes homologous to cotton in each group, further indicating that the cotton *CalSs* and the *Arabidopsis thaliana CalSs* were close in evolutionary, which was consistent with the evolutionary relationship between *Arabidopsis* and cotton. It is observed that most of the *CalSs* derived from At-subgenome of two cultivated allotetraploids cotton stayed close together with the *CalS* gene of *G. arboretum*, and the *CalSs* of Dt-subgenome stayed close together with the *CalS* gene of *G. raimondii*, which was consistent with the hypothesis that the allotetraploid cotton species were produced by the recombination of two diploid cotton species (Liu et al., 2015). Phylogenetic tree analysis suggested that the *CalS* homologous gene in cotton may have similar functions.

### Gene structure and amino acid motif analysis of the *CalSs*

**Figure 2:**
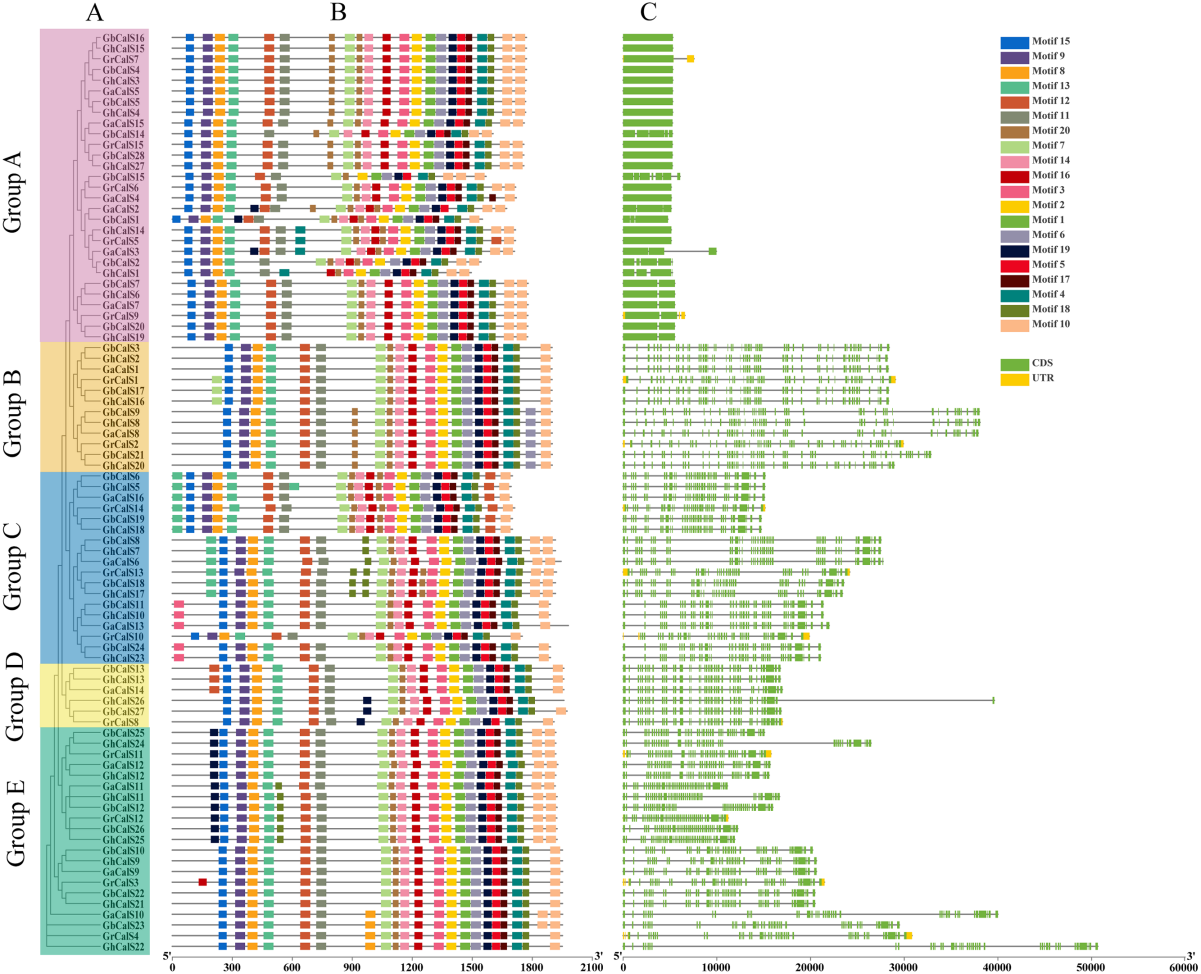
Conservative motif and exon-intron structure of *CalS* genes in cotton. (A) The evolutionary tree of *CalSs* was constructed using MEGA7. (B) Conservative motif of *CalSs*. The 20 motifs are displayed in different colored boxes. (C) Exon-intron structure of *CalSs*. Introns are presented by grey lines, exons by green boxes, and UTR for yellow boxes.

The diversity of gene structure and differences in conserved motifs are the manifestations of the evolution of multigene families (Magwanga et al., 2018). The distribution of exon/intron regions of *CalSs* was analyzed to understand the diversity of gene structure (Fig. 2). The number of *CalSs* exons varied from 1–51, and most *CalSs* had more than 35 exons (57/86, 66.2%). Clearly, these *CalSs* were divided into an exon-poor group (<7 exons, group A) and other exon-rich groups (>37 exons, group B–E) (Fig. 2, Table S2). The exons of *CalSs* had high similarity in the same group, and the number of exons in group B, group D, and group E were the same (Fig. 2, Table S2).

The motif is a conserved region in the sequence (Magwanga et al., 2018). We identified 20 possible motifs using MEME (Fig. 2). Interestingly, all *CalSs* except *GbCalS2/14/15* and *GhCalS1* contained motif1-20 and were arranged in the sequence of motif15-9-8-13-12-11-7-20-14-16-3-2-1-6-19-5-17-4-18-10. The distribution of *CalSs* were slightly different among different groups, and only the number and arrangement position were different. The number and arrangement of motifs in the same group of *CalSs* were more similar than those in other groups.

### Chromosomal location, gene duplication, and syntenic analysis of the *CalSs* in *Gossypium spp*

**Figure 3:**
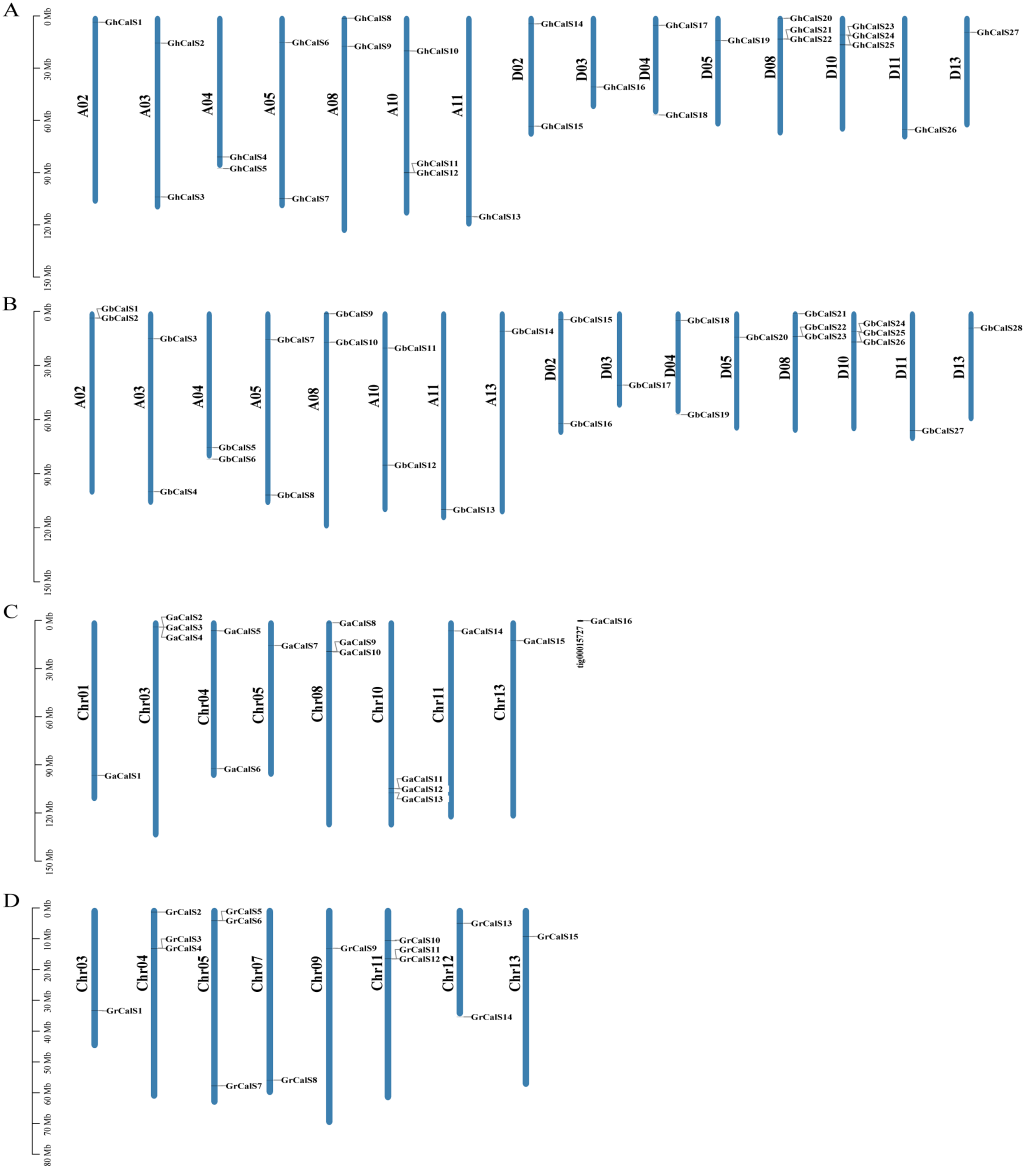
Distribution of 86 *CalSs* on cotton chromosomes. The chromosome name is on the left of each chromosome, and the gene ID is on the right. (A) *G. hirsutum*; (B) *G. barbadense*; (C) *G. arboretum*; (D) *G. raimondii*.

Based on the sequencing and annotated information of the four cotton genomes, the chromosome length and the distribution of genes on chromosome could be analyzed (Fig. 3). The distribution of *CalSs* in the two heterotetraploid cotton species chromosomes was highly similar. For example, *CalSs* had the same number and distribution on chromosomes A03, A04, A05, A08, A11. In *G. hirsutum*, 27 *GhCalSs* were distributed on 15 chromosomes, including 13 *GhCalSs* in At-subgenome and 14 *GhCalSs* in Dt-subgenome. In *G. barbadense*, 28 *GbCalSs* were distributed on 16 chromosomes, including 14 *GbCalSs* in At-subgenome and 14 *GbCalSs* in Dt-subgenome. In *G. arboreum*, 16 *GaCalSs* were distributed on eight chromosomes and a scaffold. In *G. raimondii*, 15 *GrCalSs* were distributed on eight chromosomes. Most *CalSs* occured at the upper or lower arms of chromosomes. D08 and D10 chromosomes both had the largest number of *CalSs* in the two allotetraploid cotton. Obviously, chromosome length was not positively correlated with the distribution number of *CalSs* on chromosomes.

Gene duplication is the basis for the functional differentiation of homologous genes, the main reason for the generation of new functional genes (Conant & Wolfe, 2008). In order to explain the gene replication events of *CalSs* in cotton, we identified 15, 14 paralogous gene pairs in *G. hirsutum*, *G. barbadense* respectively, and one pair in *G. arboreum*. But there was no paralogous gene pair in *G. raimondii* (Table S4). *GhCalS21/22*, *GbCalS1/2* as well as *GbCalS22/23* were tandem duplication. In the four cotton species, the duplication events of the *CalSs* were WGD/Segmental, Tandem Duplicates, Dispersed, and proximal duplication, and the main expansion mechanism was WGD/Segmental (Table S2).

**Figure 4:**
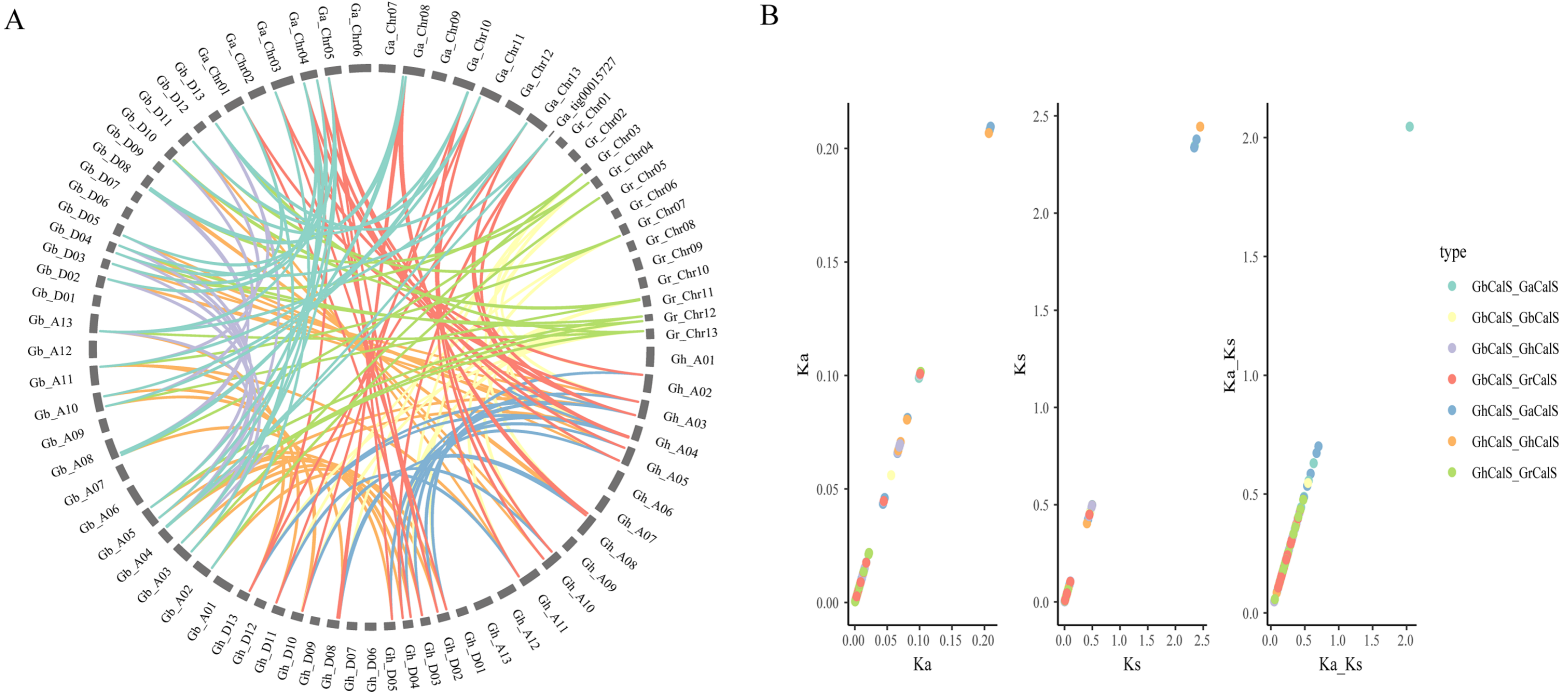
Collinearity analysis of *CalSs* in tetraploid and diploid cotton. (A) Orthologous and paralogous gene pairs among tetraploid and diploid cotton species. The lines represented by various colors indicates the syntenic regions around *CalSs*, and the color between the same species is the same (B) Ka, Ks, Ka/Ks distribution of *CalS* gene pairs. Ka, Ks, Ka/Ks analysis of *GbCalS-GaCalS*, *GbCalS-GbCalS*, *GbCalS-GhCalS*, *GbCalS-GrCalS*, *GhCalS-GaCalS*, *GhCalS-GhCalS*, *GhCalS-GrCalS*.

In order to illustrate the collinearity of *CalS* genes, we analyzed the orthologous and paralogous gene pairs (Fig. 4, Table S4). There were 31 *CalS* orthologous gene pairs among *G. arboretum* and two allotetraploid cotton species, including 15 pairs between with At-subgenome of *G. hirsutum* and 16 pairs between with At-subgenome of *G. barbadense*. There were 17 *CalS* orthologous gene pairs among *G. raimondii* and two allotetraploid cotton species, including nine pairs between with Dt-subgenome of *G. hirsutum* and eight pairs between with Dt-subgenome of *G. barbadense*. Meanwile, Ka/Ks of *CalS* homologous pairs were calculated to further understand the adaptation of the CDS region of *CalSs* (Fig. 4, Table S5). Most of the homologous gene pairs Ka/Ks < 1, and about 94.6% gene pairs had a Ka/Ks ratio less than 0.5, which meant that almost all gene pairs underwent purification selection. Only Ka/Ks > 1 of *GaCalS2/GbCalS1* indicated that this was a positive selection for beneficial mutations.

### Analysis of Cis-acting elements in promoter

**Figure 5:**
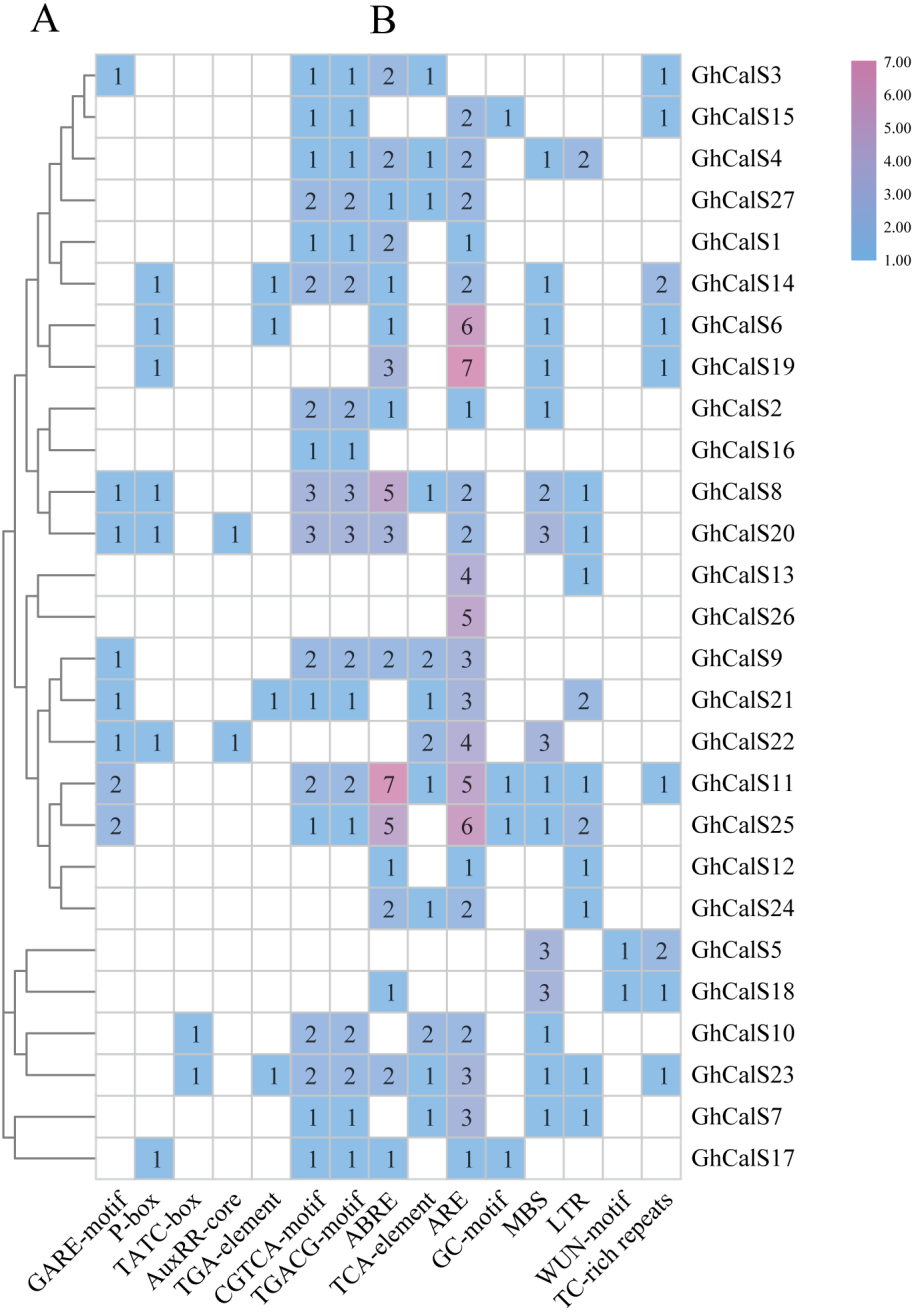
Cis-acting elements on promoters of the *GhCalSs*. (A) The evolutionary tree of *GhCalSs* was constructed wsing MEGA7. (B) The cis-acting element on the promoter of *GhCalSs*. Number of each cis-acting element in the promoter region.

Transcription factors can be combined with cis-elements in the promoter region to regulate gene transcription. Investigation of upstream regulatory sequence can help us to well understand the regulation mechanism and also supportive to estimate the potential function of the gene (Fig. 5, Table S6). Given the effect of plant hormones in abiotic stress, we focused on plant hormone responsive elements in promoter regions. ABA- (ABRE), auxin- (AuxRR-core, TGA-element), Gibberellin- (GARE-motif, P-box, TATC-box), MeJA- (CGTCA-motif, TGACG-motif), SA- (TCA-element) responsive elements were found in the promoters of 18, 6, 14, 18, 12 *GhCalSs*. All *GhCalSs* contained hormone response elements except the *GhCalSs* in Group D and *GhCalS5* in Group C. More than half of the *GhCalSs* contained ABA/GA/MeJA-responsive elements. Auxin-responsive elements only in *GhCalS6/14/20/21/22/23*. Meanwhile, we also paid attention to elements related to stress. Low-temperature- (LTR), wound- (WUN-motif), drought- (MBS), stress- (TC-rich repeats), anaerobic induced response element (ARE), anoxic specific inducibility element (GC-motif) were found in the promoters of 11, 2, 15, 9, 23, 4 *GhCalSs*. Wound-responsive elements only in *GhCalS5* and *GhCalS18*. Anoxic specific inducibility elements only in *GhCalS11/15/17/25*. In addition, these results suggested that *CalSs* might regulated by hormone and abiotic stresses.

### Expression patterns of the *GhCalSs* under abiotic stresses

**Figure 6:**
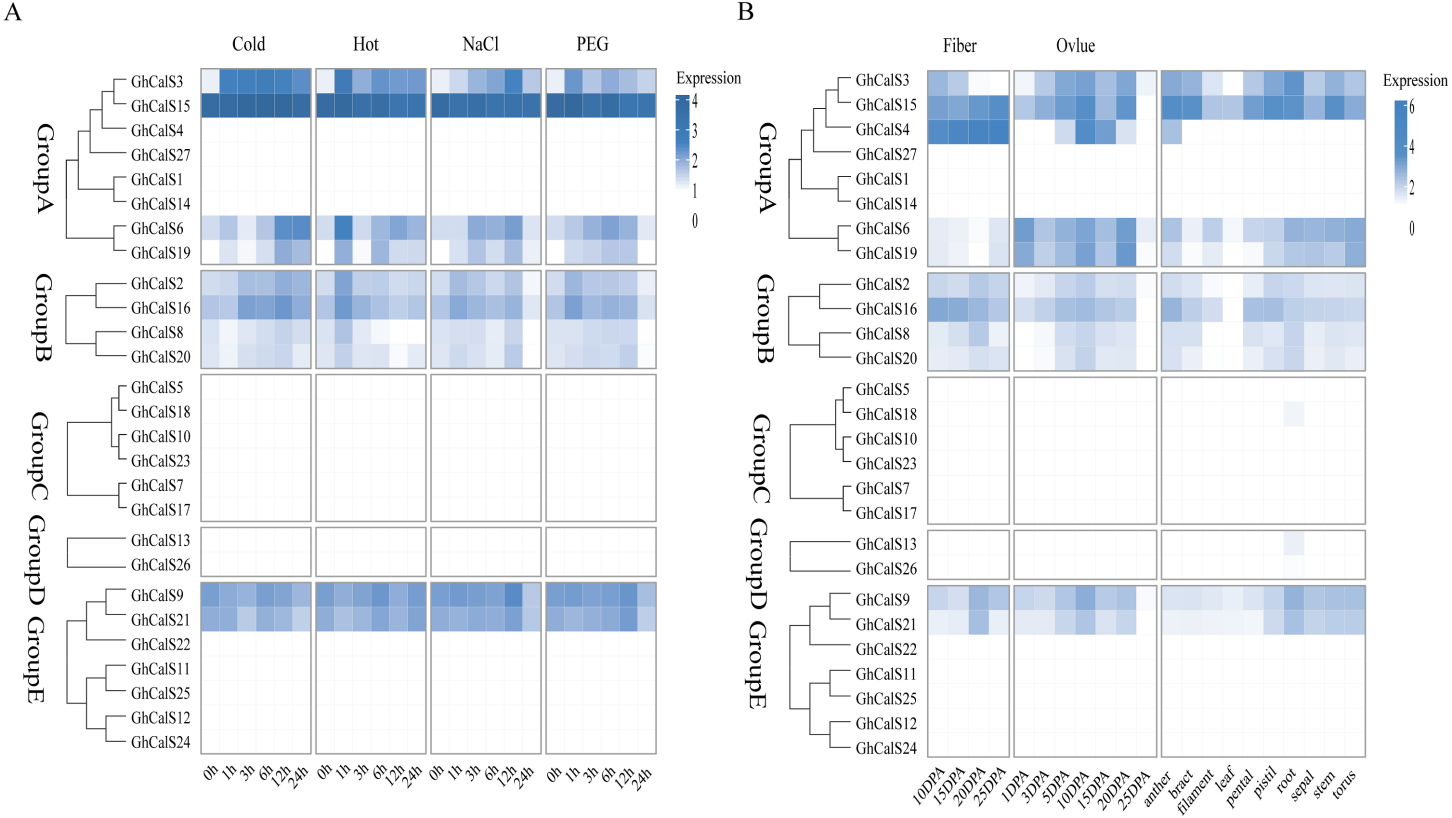
RNA sequence profiling of the *CalS* gene family. (A) Heatmap displaying expression of expressed *GhCalSs* under hot, cold, NaCl, and PEG treatment (B) Heatmap displaying expression of expressed *GhCalSs* in each tissue.

**Figure 7:**
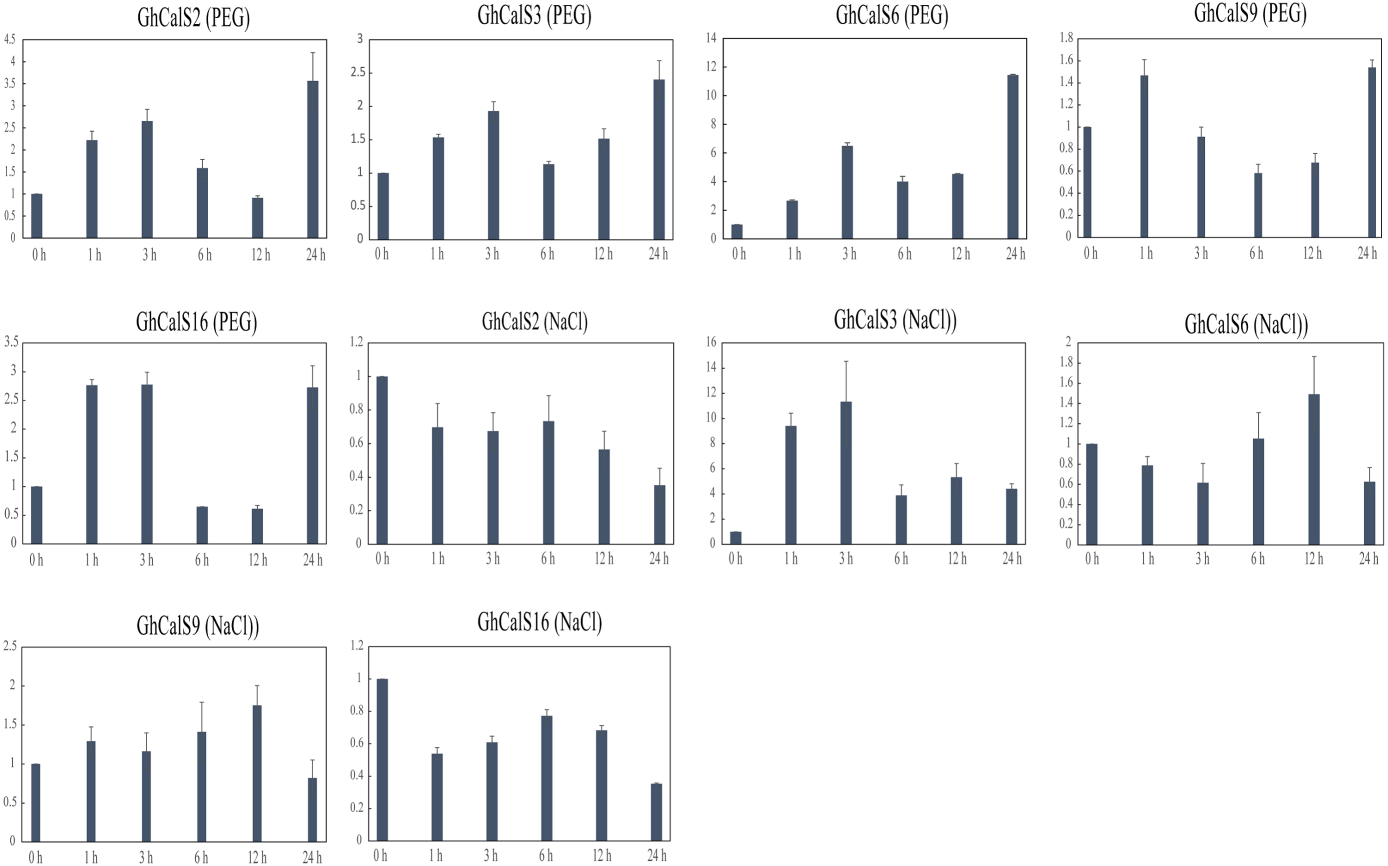
qRT-PCR results of *GhCalSs* under PEG and NaCl.

Previous studies have reported that the *CalSs* respond to abiotic stresses (Cui & Lee, 2016; Jacobs et al., 2003; Fromm et al., 2013). To understand the response of *GhCalSs*, we used public RNA-seq data of TM-1 treated with cold, hot, NaCl, and PEG to observe the expression patterns of *GhCalSs* (Fig. 6). Interestingly, all expressed *GhCalSs* were induced by different abiotic stresses, and the expression patterns were different. The expression of *GhCalS3* was significantly up-regulated under cold, hot, NaCl, and PEG. The expression patterns of *GhCalSs* in the same group were slightly consistent, such as *GhCalS3* and *GhCalS6*, *GhCalS2* and *GhCalS16*. In order to verify the results obtained by the above transcriptome, cotton seedlings were treated with PEG and NaCl, and then the *GhCalS2/3/6/9/16* were selected for qRT-PCR (Fig. 7). The expression of *GhCalS3* and *GhCalS6* in Group A were up-regulated within 24 h under PEG treatment and reached the peak at 24 h. The expression of *GhCalS2*, *GhCalS9*, *GhCalS16* were up-regulated at first and then down-regulated and last up-regulated after PEG treatment. After NaCl treatment, there was no consistent trend of gene expression. *GhCalS3* was significantly induced by NaCl and significantly up-regulated at 3 h. Both *GhCalS2* and *GhCalS16* of Group B were down-regulated within 24 h. The expression of *GhCalS6* and *GhCalS9* reached a peak at 12 h. These findings indicated that the expression patterns of several *GhCalSs* were changed after treatment, which proved that *GhCalSs* increased adaptability to abiotic stress (Fig. 6).

### Enrichment analysis of the *GhCalSs*

**Figure 8:**
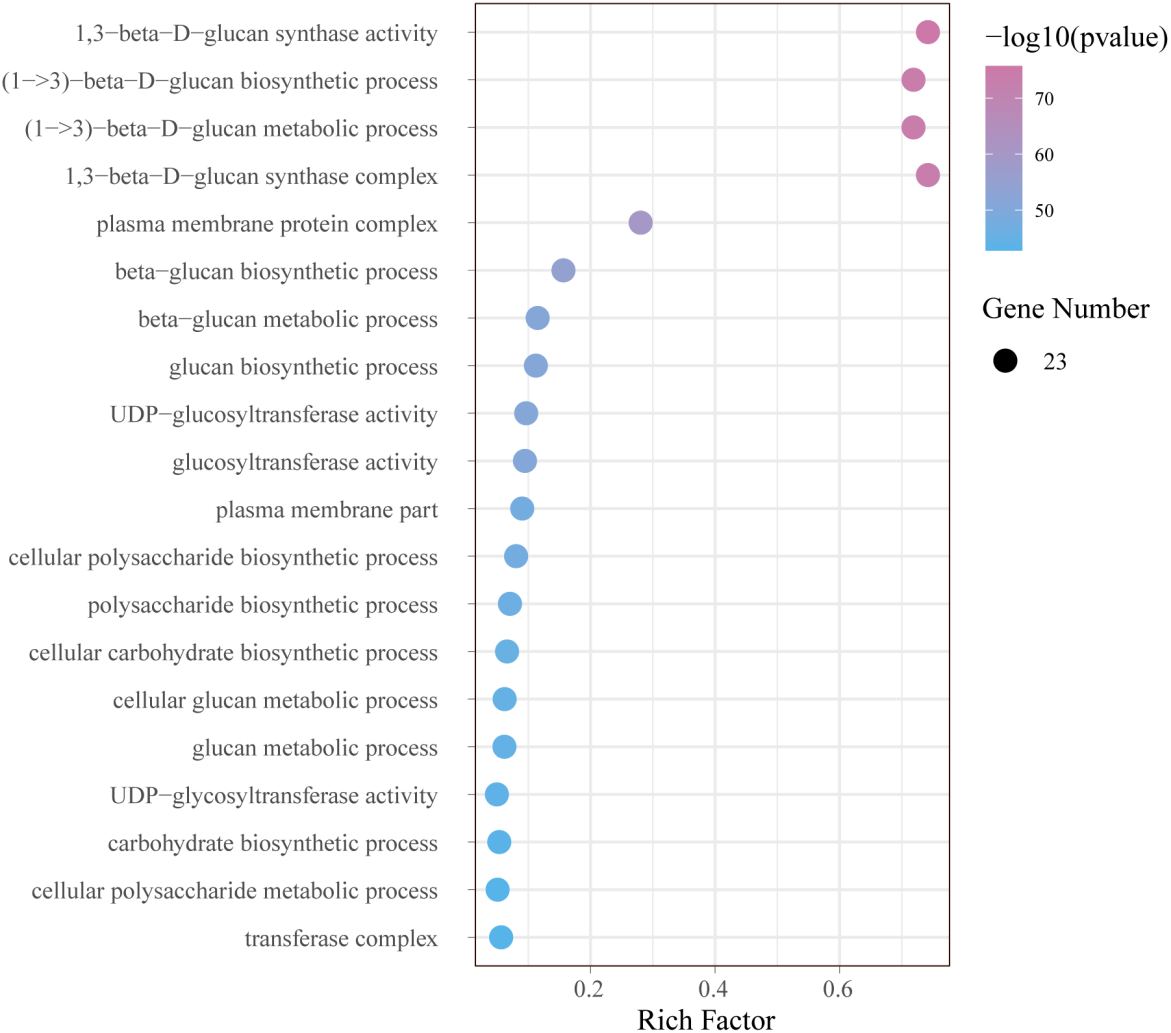
Bubble plot showing GO enrichment analysis of *GhCalSs*. The top 20 GO terms significantly enriched by *GhCalSs*.

In order to further understand the function of *GhCalSs*, we carried out functional enrichment annotation of gene ontology (GO) using pvalue of ≤ 0.05 as the cutoff. The results improved our accurate understanding of gene function, including many significantly enriched terms (Fig. 8, Table S7). The GO-BP enrichment results showed 34 terms such as (1->3)-beta-D-glucan biosynthetic process (GO:0006075), beta-glucan metabolic process (GO:0051273), cellular carbohydrate biosynthetic process (GO:0034637), cellular macromolecule biosynthetic process (GO:0034645). The GO-CC enrichment results discovered 16 terms such as 1,3-beta-D-glucan synthase complex (GO:0000148), plasma membrane protein complex (GO:0098797), transferase complex (GO:1990234), catalytic complex (GO:1902494). The CC terms enriched by GO were consistent with the subcellular localization of *GhCalSs*. GO-MF enrichment exposed 8 terms, including 1,3-beta-D-glucan synthase activity (GO:0003843), UDP-glucosyltransferase activity (GO:0035251), catalytic activity (GO:0003824), hexosyltransferase activity (GO:0016758). In short, the GO enrichment results confirmed the function of the *GhCalSs* in many biological processes, which were associated with 1,3-β-D-glucan synthetic activity, hydrolyzase activity, and membrane parts.

### *GhCalSs* expression patterns in various tissues and their role in fiber development

We used transcriptome data from different tissues of *GhCalSs* to gain insight the tissue-specific expression patterns of cotton. For instance, *GhCalSs* were expressed in various tissues, and some of them were highly expressed. Some of *GhCalSs* were expressed in one or more tissues (*GhCalS2, 3, 4, 6, 8, 9, 15, 16, 19, 20, 21*). However, the expression of a few genes (*GhCalS1, 5, 7, 10, 11, 12, 13, 14, 17, 18, 22, 23, 24, 25, 26, 27*) did not show any expression in any tissues.

**Figure 9:**
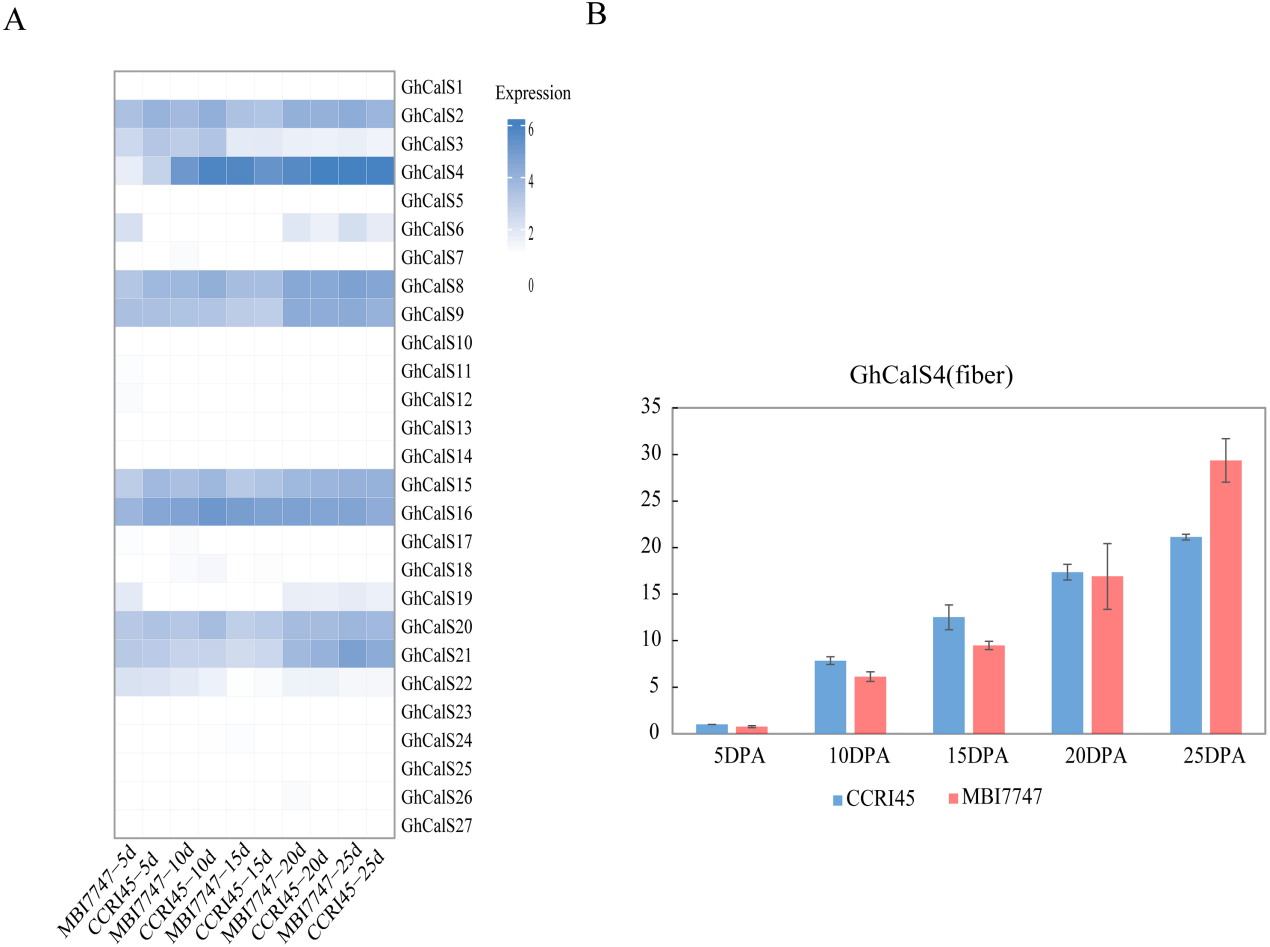
Expression patterns of *GhCalSs* in cotton fiber. (A) The expression of *GhCalSs* of MBI7747 and CCRI45 at different fiber developmental stages. (B) qRT-PCR results of *GhCalS4* at different fiber developmental stages.

In order to determine the effect of *GhCalSs* in cotton fiber development, we focused on the expression of *GhCalSs* in different fiber developmental stages of two samples, MBI7747 and CCRI45, with different lengths and strengths (Lu et al., 2017) (Fig. 9). The expression of *GhCalS4* was the highest in TM-1, MBI7747, CCRI45 fiber tissue, so it was speculated that *GhCalS4* had an important function in cotton fiber development. In order to further determine its function in fiber development, qRT-PCR was used to analyze the *GhCalS4* expression differences in two samples (Fig. 9). The results showed that the expression level of *GhCalS4* in the two samples gradually increased from 5 DPA to 25 DPA, which was consistent with the transcriptome data of TM-1 used above. The expression of *GhCalS4* in CCRI45 was higher than in MBI7747 at 5DPA, 10DPA, 15DPA but was significantly lower than that of MBI7747 at 25DPA (Lu et al., 2017). Thus, *GhCalS4* may be involved in cotton fiber elongation.

## Discussion

Callose plays a vital role in plant growth, development, and resistance to various adverse factors (Piršelová & Matušíková, 2012). The gene family of *callose synthase* has been identified in a variety of plants. In this study, we identified the *CalSs* in *G. hirsutum*, *G. barbadense*, *G. raimondii*, and *G. arboreum*, aiming to understand the role of *CalS* family in the cotton development.

A total of 86 *CalSs* were identified in four cotton species. They were divided into five groups based on evolutionary relationships. we divided *CalSs* (*AtCalS9-12* homologous) into two groups due to the large difference of the CDS number, and the other groups were the same as those in *Arabidopsis thaliana*. The *CalSs* number was 2:2:1:1 in group B/C/D, which was consistent with the evolutionary relationship among cotton species (Table S3). Compared with *Arabidopsis thaliana*, different percentages existed between subgroups. The percentages of Group A and Group E were significantly different from those of the corresponding *CalSs* in *Arabidopsis thaliana*, suggesting that these genes in cotton may have functional differences with homologous genes in *Arabidopsis thaliana* to a certain extent. These results will help to validate the function of cotton *CalS* homologous gene with *Arabidopsis thaliana*.

Tetraploid cotton species are formed by natural crossbreeding between *G. raimondii* and *G. arboretum* (Wendel et al., 2009). Thus, the four cotton species are closely related in evolution. In Fig. 4, the orthologous gene pairs of *CalSs* were all clustered in the same branch or group. Phylogenetic and orthologous genes of *CalS* further indicated that the results of this study were consistent with the evolutionary view.

A large number of hormone-responsive elements were identified on *GhCalSs* promoters which may be involved in the regulation of *GhCalSs*. Salicylic acid (SA) was an endogenous signal molecule in plants (Loake & Grant, 2007). In *Arabidopsis thaliana*, the expressions of *AtCalS1/5/9/10/12* were up-regulated by exogenous SA. Abscisic acid (ABA) played an important part in coping with a variety of adverse factors, closely related to callose synthesis (Liu et al., 2017). During the dormancy of *Populus tomentosa* buds, short-day induced ABA biosynthesis, promoted the expression of *PtCalS1*, callose deposited at the plasmodesmata to form blockage, which prevented the growth signal molecules from entering the cell and kept the dormancy state of buds (Tylewicz et al., 2018). Jasmonic acid (JA) was also involved in callose regulation, and Methyl Jasmonate (MeJA) application promoted callose deposition in grape leaves. Inhibition of the expression of *Cationic peroxidase 3* (*OCP3*), a negative regulator of the JA pathway, increased callose deposition (Repka, Fischerová & Šilhárová, 2004). In conclusion, ABA, JA, and SA were involved in the regulation of callose deposition. *GhCalSs* promoters with ABA, SA and JA response elements were highly likely to be regulated by them in cotton. However, how *CalS* gene is regulated by these hormones in the face of biotic-abiotic stress or growth and development is not known, which needs to be further studied.

Callose deposition is one of a series of coping strategies in plants to abiotic stress. Low temperature stimulation of *maize* leaves increased callose content and reduced transport of photosynthate in phloem (Wu et al., 2018). In *Arabidopsis thaliana*, *AtCalS7*, *AtCalS8* and *AtCalS12* were associated with callose synthesis under the condition of wound (Jacobs et al., 2003; Cui & Lee, 2016; Xie et al., 2011). In this study, public transcriptome data were used to analyze the responses of cotton leaves to cold, heat, salt and drought, and qRT-PCR was used to verify the results, which showed that *CalSs* were involved in abiotic stresses.

Callose deposits regulate material transport and control plant development. In this study, *GhCalS4* was highly expressed in fibers and differentially expressed in MBI7747 and CCRI45 fibers at each fiber developmental stage (5/10/15/20/25 DPA). It has been reported that callose deposition may be involved in the closure of plasmodesmata, and the closure of plasmodesmata had an important function in the elongation of cotton fibers (Ruan et al., 2004). In Sea Island cotton, plasmodesmata remain open longer than in Upland cotton, allowing sucrose to be fed into fibroblasts, which eventually increase osmotic potential by hydrolysis to fructose and glucose. The more soluble sugar, K^+^ accumulated, the higher the cell leavening pressure, which promoted the elongation of cotton fiber (Hu et al., 2019). MBI7747 is a chromosome segment substitution line (CSSL) with different genetic background constructed by crosses between the upland cotton CCRI45 as the recurrent parent and the Sea Island cotton Hai 1 with outstanding fiber quality through the combination of high-generation backcrossing and molecular marker-assisted selection. The fiber length and strength of MBI7747 are better than CCRI45. In CCRI45, the expression level of this gene at 5DPA, 10DPA and 15DPA were all higher than those of MBI7747 during fiber elongation, and it was speculated that the degree of callose deposition in MBI7747 was lower than that of CCRI45, which made more sucrose input into fiber cells to increase osmotic potential and promote fiber elongation. Thus, *GhCalS4* may be an introgression gene or there was difference in epigenetic regulation.

## Conclusions

In this study, we identified 86 *CalSs* from *G. hirsutum*, *G. barbadense*, *G. raimondii*, and *G. arboreum* using conserved domains. Phylogeny, gene structure, motif, chromosome location and homologous genes were analyzed. It indicated that *CalSs* have been highly conserved during evolution by the analysis of *CalSs* structure, conversed motifs, and syntenic blocks. WGD/Segmental replication was the main driving force for the amplification of *CalS* family in cotton, and purification selection played an important role in the evolution of *CalSs*. In addition, the cis-acting elements of *GhCalSs* related to hormone regulation and development and their expression patterns in stresses and tissues were also analyzed. *CalS* gene can be induced by abiotic stress. Furthermore, the expression difference of *GhCalS4* in fiber of different length and strength materials was analyzed. It was speculated that *GhCalS4* played a major role in fiber elongation. These findings could lay a foundation for further study on the role of *CalS* gene in stress response and fiber development.

## Supplemental Information

10.7717/peerj.12557/supp-1 Supplemental Information 1 Primers used in this study

10.7717/peerj.12557/supp-2 Supplemental Information 2 List of the identified *CalS* genes in cotton

10.7717/peerj.12557/supp-3 Supplemental Information 3 List of the number of *CalS* family genes

10.7717/peerj.12557/supp-4 Supplemental Information 4 Orthologous and paralogous *CalS* gene pairs among *G. hirsutum, G. barbadense, G. raimondii, and G. arboretum.*

10.7717/peerj.12557/supp-5 Supplemental Information 5 Distribution of Ka, Ks, Ka/Ks of *CalS* gene pairs

10.7717/peerj.12557/supp-6 Supplemental Information 6 Cis-acting elements in the promoter regions of *GhCalSs.*

10.7717/peerj.12557/supp-7 Supplemental Information 7 The GO enrichment analysis of *GhCalSs*

10.7717/peerj.12557/supp-8 Supplemental Information 8 Raw data: qRT-PCR results of GhCalSs under salt, drought, and different fiber development stages

## Additional Information and Declarations

### Data Availability

The following information was supplied regarding data availability: The raw data are available in the Supplemental File.
